# The Coupled Photothermal Reaction and Transport in a Laser Additive Metal Nanolayer Simultaneous Synthesis and Pattering for Flexible Electronics

**DOI:** 10.3390/nano6010012

**Published:** 2016-01-08

**Authors:** Song-Ling Tsai, Yi-Kai Liu, Heng Pan, Chien-Hung Liu, Ming-Tsang Lee

**Affiliations:** 1 Department of Mechanical Engineering, National Chung Hsing University, Taichung 402, Taiwan; s3677265@gmail.com (S.-L.T.); kevin5053@livemail.tw (Y.-K.L.); carus@nchu.edu.tw (C.-H.L.); 2 Department of Mechanical and Aerospace Engineering, Missouri University of Science and Technology, Rolla, MO 65409, USA; hp5c7@mst.edu

**Keywords:** laser direct write, flexible electronics, reactive silver ink, laser direct synthesis and patterning, additive microfabrication

## Abstract

The Laser Direct Synthesis and Patterning (LDSP) technology has advantages in terms of processing time and cost compared to nanomaterials-based laser additive microfabrication processes. In LDSP, a scanning laser on the substrate surface induces chemical reactions in the reactive liquid solution and selectively deposits target material in a preselected pattern on the substrate. In this study, we experimentally investigated the effect of the processing parameters and type and concentration of the additive solvent on the properties and growth rate of the resulting metal film fabricated by this LDSP technology. It was shown that reactive metal ion solutions with substantial viscosity yield metal films with superior physical properties. A numerical analysis was also carried out the first time to investigate the coupled opto-thermo-fluidic transport phenomena and the effects on the metal film growth rate. To complete the simulation, the optical properties of the LDSP deposited metal film with a variety of thicknesses were measured. The characteristics of the temperature field and the thermally induced flow associated with the moving heat source are discussed. It was shown that the processing temperature range of the LDSP is from 330 to 390 K. A semi-empirical model for estimating the metal film growth rate using this process was developed based on these results. From the experimental and numerical results, it is seen that, owing to the increased reflectivity of the silver film as its thickness increases, the growth rate decreases gradually from about 40 nm at initial to 10 nm per laser scan after ten scans. This self-controlling effect of LDSP process controls the thickness and improves the uniformity of the fabricated metal film. The growth rate and resulting thickness of the metal film can also be regulated by adjustment of the processing parameters, and thus can be utilized for controllable additive nano/microfabrication.

## 1. Introduction

The development of rapid and cost-effective nano/microfabrication processes for electric circuitry on flexible substrates has gained significant attention as a pathway to low cost, large area, flexible and wearable electronics [1]. Conventional micro-fabrication processes (such as photolithography) require complex, sophisticated and expensive equipment, and the process often employs toxic chemicals as etchants and developers. In addition, a general microfabrication process is not suitable for use on flexible material, because the corrosion resistance of most flexible materials is not high and they are also intolerant to the elevated temperatures often encountered in a standard photolithography process. In addition, the manufacture of circuit patterns using photolithography often requires the use of a mask to transfer the pattern to the substrate. It is very difficult to make changes to a mask once it has been prepared, and this places further limitations on a process which is already very expensive and time-consuming. This has resulted in much attention being given to the development of low cost micro-electromechanical manufacturing technology that can be used for the manufacture of microscale electronics on a flexible substrate as required, the design of which can be very easily and quickly changed [2,3].

In recent years, the research and technology development of processes using nanomaterials has attracted the attention of many researchers owing to several unique properties of the nanomaterial, especially on the size dependent melting temperature reduction [4]. The characteristics of low melting temperature nanomaterials has been successfully applied in several non-conventional nano/microfabrication techniques such as laser direct writing [5,6], inkjet printing [7,8], nanoimprinting [9], the low temperature welding of nanowire percolations [10], and the low temperature sintering of nanoparticles [11,12]. The main objectives of the development of advanced microscale manufacturing processes by the use of nanomaterials are the realization of large area, fast, non-vacuum and mask-free microscale manufacturing technology with a high degree of design flexibility. Nevertheless, the requirement of nanomaterials in these techniques significantly limits the application of such approach due to the costly and complex process of nanomaterial synthesis, as well as the instability of most nanomaterials in a normal environment. Therefore, low temperature, nanoparticle-free processes that are compatible to flexible substrates are more ideal for fabricating flexible electronics [13]. In this study, we applied and revised a laser direct synthesis and patterning (LDSP) technology [3,14] to replace microfabrication that involves masks, vacuum systems and nanomaterials. In the process, a transparent and particle-free silver ion reaction solution is applied on the surface of a polymer substrate (polyimide film). The beam from a continuous wave (CW) laser, focused on the surface of the substrate to achieve rapid and localized heating, is guided by a galvanometer scanning system. Silver ions are selectively reacted and precipitated on the substrate surface to form the circuit pattern. This process, which can be carried out at normal room temperature and pressure, is cheaper than other micro-fabrication processes and there is no need for the synthesis of expensive nanomaterials. Our experiments have produced excellent stabilized silver nano-microstructure patterns on polymer substrates. In this study, we further experimentally investigated the effect of the additive solvent on the resulting silver nanostructure. In addition, from the experimental results, we found the transient thermal effect near the laser focal spot to be of vital importance. However, reports on the analysis of the transport characteristics of the laser thermal additive nano/microfabrication processes are still rather limited [6,15,16,17,18], and the fluid flow effects were often neglected. Especially for the LDSP, our literature survey revealed no published studies of the opto-thermo-fluidic transport phenomena coupled with chemical reaction rate analysis in this process. However, as also to be shown in the results of this study, the thermally induced fluid flow is very important to the growth rate and stability of laser additive microfabrication in reactive liquid environment [19,20]. To address this deficiency in theoretical study we conducted experiments and numerical simulation to investigate the effects of process parameters on the growth rate of the resulting silver nanostructures. One important goal was to further the understanding of the impact of various processing parameters (laser power, scanning speed and solution concentration *etc.*) on LDSP fabrication. It is emphasized that the results of this experimental and numerical investigation were aimed to serve as reference for the subsequent improvement of LDSP technology and other advanced laser additive nano/microfabrication processes with similar configurations and physics.

## 2. Experimental

**Figure 1:**
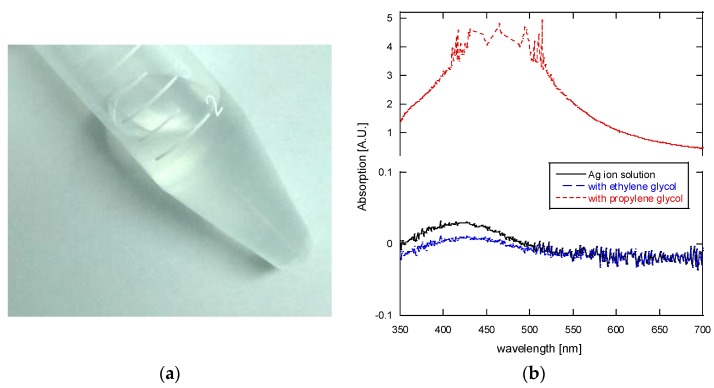
(**a**) The silver ion reaction solution used in this study; and (**b**) The absorbance spectrum of the silver ion solution and its mixture (1:1) with ethylene glycol or propylene glycol.

The silver ion solution (ink) used in this study was prepared based on the procedure discussed in the referenced articles. [14,21]. In brief, 1 g of silver acetate (silver acetate, anhydrous 99%, Alfa Aesar, Lancashire, UK) was dissolved in 2.5 mL of aqueous ammonium hydroxide (28%–30%, JT Baker, Center Valley, PA, USA), followed immediately by stirring and the addition of 0.2 mL of formic acid (88%, JT Baker, Center Valley, PA, USA). The ink was allowed to settle for 12 h in refrigerator at about 4 °C to allow the larger silver particles to precipitate on the bottom of the container. Note that the addition of formic acid results in an exothermic reaction and should be carried out in an ice bath to prevent an excessively fast reaction which will affect the concentration of the silver ion solution. The prepared silver ion solution is a clear liquid as shown in Figure 1 and the UV-Vis absorbance spectrum measurement (Genesys 10S UV-Vis Spectrophotometer, Thermo Scientific, Waltham, MA, USA), also shown in the figure, confirms that the ion solution is free of silver nanoparticles. Note that the slight negative values shown in the spectra are due to the resolution limit of the spectrophotometer. This silver ion solution is mixed with ethylene glycol (JT Baker, Center Valley, PA, USA) or propylene glycol (JT Baker, Center Valley, PA, USA) at 1:1 (by volume) to prepare the processing solution for the LDSP. It is emphasized, as will be seen in the results and discussion section, that the addition of proper glycol solvent is essential to the attainment of stable results and good quality silver patterns.

**Figure 2:**
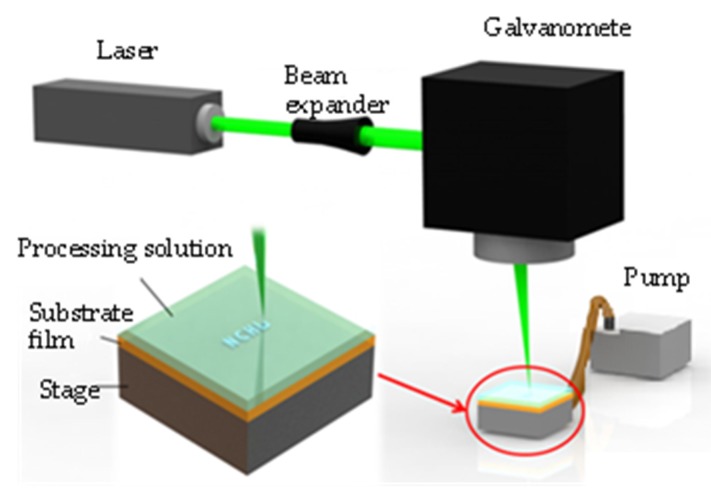
Experimental apparatus of laser direct synthesis and patterning (LDSP).

A polyimide sheet (100 μm thick) was cleaned by rinsing with acetone, ethanol, isopropanol and methanol, consecutively. After air dried, the top surface of the sheet was treated with oxygen plasma (Atmospheric Pressure Plasma Cleaner, APPC103C, Solar Energy Tech. Inc., Taiwan. Output voltage: 10,000–50,000 V. Output frequency: 4–5 MHz) for 10 s to improve adhesion of the silver to the polyimide surface [14,19]. The polyimide sheet was then held on a vacuum suction sample holder to ensure a flat surface. A small amount of the processing solution was prepared and dispensed to form a liquid film that covered the surface of the polyimide sheet. Typically, a layer about 2 mm thick was enough to prevent the processing solution from drying out during the experiment. The laser light source used in the experiment was a green (*λ* = 532 nm) CW laser. A beam expander was used to enlarge the collimated beam to enhance focusing. A laser galvanometer scanner (SCANLAB hurryScan^®^ II-7, SCANLAB AG, Munich, Germany) was used to focus and guide the laser beam, according to a programmed pattern, onto the substrate surface. The experimental LDSP system is shown in Figure 2. The polyimide substrate absorbs the irradiated laser light and converts it to heat. The processing solution near the polyimide surface, and in the path of the beam, is heated and a highly localized reaction takes place to yield silver which is directly deposited on the substrate surface. After the laser scanning process is completed, the polyimide surface is rinsed with deionized water and then ethanol to remove unreacted silver ink [22].

To evaluate the electrical property of the resulting silver pattern, silver patterns of 1 mm (width) × 10 mm (length) were fabricated using the setup described. Note that the width of single line scan is approximately 50 μm. Therefore, to fabricate a silver line pattern with 1 mm width, the laser scans were repeated in a zig-zag fashion with a 50 μm line-to-line pitch. More details for the resolution and line width can be found in our previous report [14]. The electrical resistance of the silver lines was measured with an impedance measuring instrument (Hioki RM3544-01, range 30 mΩ–3 MΩ, accuracy ± 0.02%, HIOKI E.E. Corporation, Nagano, Japan). A white light interferometer (Zygo NV7100, resolution 0.1 nm, Zygo Corporation, Middlefield, CT, USA) was used to measure the thickness of the silver lines. In addition, to formulate the numerical simulation model, a lab-constructed and validated hemispherical radiation measurement system (facility in Prof. Yu-Bin Chen’s Lab in the Department of Mechanical Engineering, National Cheng Kung University, Tainan City, Taiwan) [23] (wavelength range: 400–1800 nm, resolution: 20 nm) was used to measure the reflectivity and transmissivity of the silver microstructure.

## 3. Numerical Simulation

**Figure 3:**
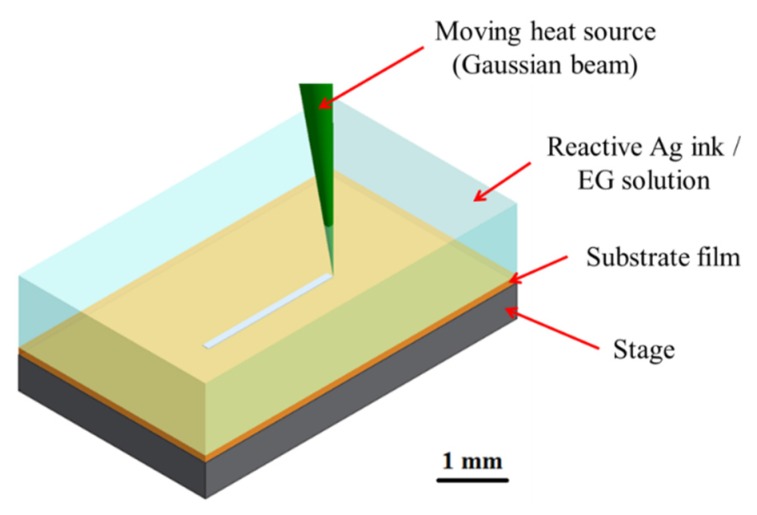
A schematic of the simulation domain used in the current study.

COMSOL^®^ Multiphysics finite element software (COMSOL Inc., Burlington, MA, USA) was used for numerical simulation to elucidate the influence of process parameters on the transport phenomena in the vicinity of the focused laser spot during the LDSP process, and also to investigate their coupled opto-thermal-fluidic effects on the silver film formation reaction. The simulation domain is shown in Figure 3. The size of the simulation domain was determined by the confirmation (from the test results) that it would not affect the simulation results, *i.e.*, there would be no edge effects. The standard continuity, momentum, and energy equations for slightly compressible fluid (where the fluid density is a function of temperature) flow were solved to obtain the temperature and fluid velocity distributions considering the buoyancy effects in the reaction fluid associated with the temperature gradient induced by heat from the moving laser beam. The heat source Q_abs_ corresponding to the absorbed energy of the moving laser spot with a speed U is given as [16,24]:

(1) $$Q_{abs}=\left(1-R\right)\gamma I_{0}exp\left[-{\left(\frac{x-Ut}{\omega }\right)}^{2}-{\left(\frac{y}{\omega }\right)}^{2}-\gamma z\right]$$

where *R* is the reflectivity, γ is the absorption coefficient, *I*_0_ is the laser intensity and ω is the size of the laser beam on the substrate surface.

As will be discussed later, a semi-empirical model for the temperature dependent growth rate of the silver line thickness was then developed using the numerical simulation results for temperature and the experimental results for silver line thickness of the benchmark case (ethylene glycol based solution, laser power: 200 mW, scanning speed: 20 mm/s). It should be pointed out that the reflectivity of the substrate increases with the thickness of the silver line. Therefore, a correlation for the reflectivity of the substrate surface covered by a silver film with different thicknesses was determined from the representative measured results (to be shown later). The correlation for the reflectivity with respect to the silver film thickness was then incorporated in the numerical analysis to determine the absorption of irradiated laser light by the polyimide substrate which will vary depending on the thickness of the silver. Testing of the stability of the numerical solution to mesh size was performed with element (grid) numbers of 793,938, 1,783,174, 3,103,532, 8,599,826 and 9,399,321, respectively. It was found that, in the computed results, the maximum temperature on the substrate surface (at the center of the laser beam) begins to converge and stabilized with a number of elements greater than 8,599,826 and a mesh system with this number of elements was therefore used for the subsequent simulations.

## 4. Results and Discussion

### 4.1. Experimental Results

**Figure 4:**
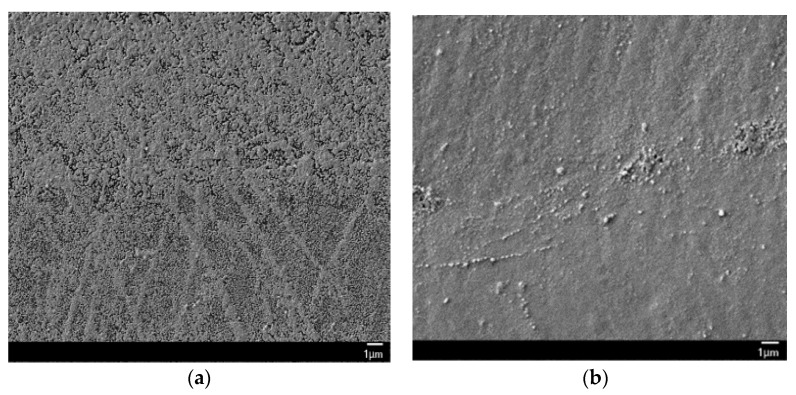
SEM images of the silver film fabricated from the reaction solution with (**a**) ethylene glycol; (**b**) propylene glycol.

Figure 4 shows a comparison of the surface structure of silver lines fabricated from reactive ion solutions mixed with ethylene glycol or propylene glycol at 1:1 (by volume). The laser power was 200 mW at a scan speed of 20 mm/s, and 10 scans were used in both cases. It can be seen that the surface of the silver deposited from solution in the propylene glycol case is smoother and less fragmentary than that from the ethylene glycol mixed solution. This is possibly due to the viscosity of propylene glycol (42cp at STP) which is higher than that of ethylene glycol (16cp at STP). In our previous study [14], it was found that the morphology of the metal line fabricated by LDSP is seriously affected by the ratio of the added ethylene glycol to aqueous ammonium hydroxide, where an increase in the ratio of ethylene glycol results in straighter and more well-defined silver lines. This was attributed to the higher boiling point and viscosity which inhibits vaporization and reduces flow of the reaction solution near the laser focus point. Since ethylene glycol and propylene glycol have a similar boiling temperature, we conclude that the improvement in structure (smoother and less porous) of the silver lines is the result of the higher viscosity of propylene glycol.

The line width resolution is significantly affected by the concentration of the reactive ion solution as previously investigated and reported [14]. Generally, to attain a silver line with better resolution and line width control, a reactive ion solution with higher ethylene glycol (EG) concentration should be used. However, this would decrease the growth rate of the silver line in consequence. Thus, in the current study, we used the reactive solution with ink to EG ratio equals to 1:1. It should also be pointed out that the line edges fabricated by the LDSP process are wider, thicker and rougher, owing to the deceleration of the laser scans close to the edges as was illustrated in our previous experiment report [22]. One possible method to resolve this issue is adjusting the laser power at locations where the scanning speed is accelerating or decelerating. In the current study, however, we focused on investigating the transport phenomena and line properties in the section with stable processing parameters.

**Figure 5:**
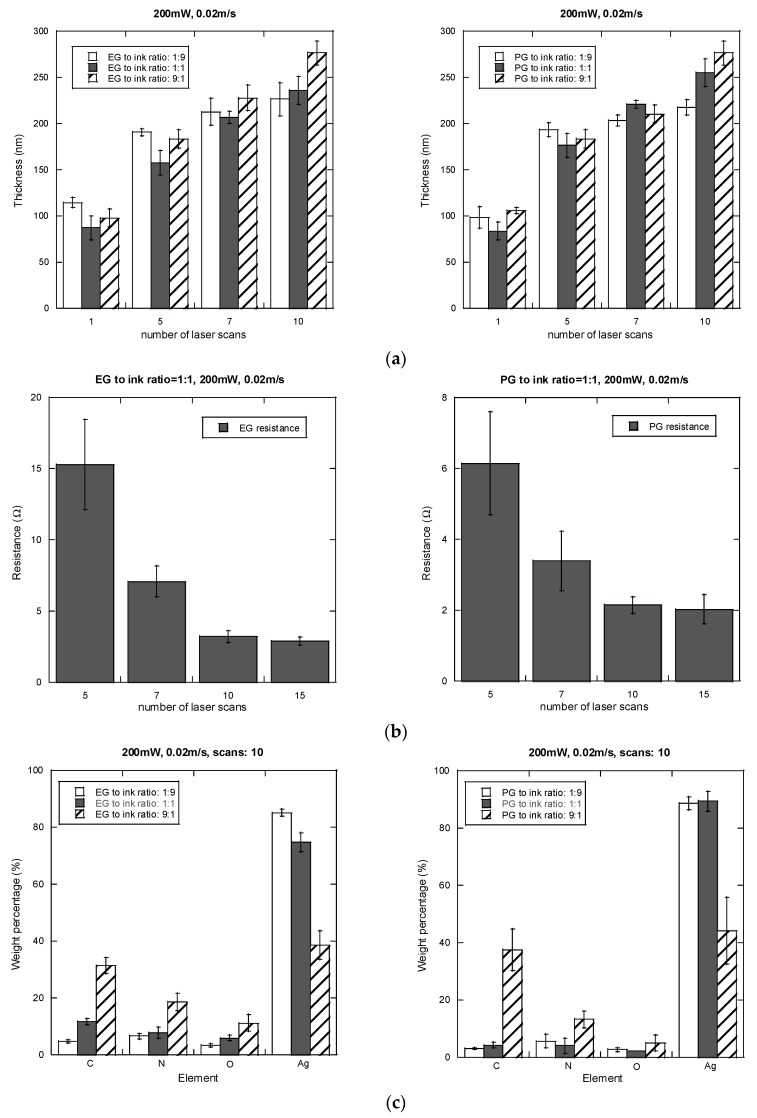
(**a**) Thickness and (**b**) Electrical resistance of the silver films with respect to the number of laser scans; (**c**) EDS results for the silver lines with 10 scans.

Figure 5a,b show the variations in thickness and electrical resistance of the silver lines with respect to the number of scans. The electrical resistance decreases with an increase in film thickness as the number of scans goes up until the values become saturated with more than ten laser scans. These results are similar to those were reported for the EG based solution where the resistance of the silver lines fabricated using LDSP is saturated after about ten scans [14]. Thus, other measurements on the line thickness, reflectivity and element ratio analysis in the current study were conducted for silver lines up to ten laser scans. The electrical resistance of silver lines fabricated with propylene glycol additive is less than that with ethylene glycol if fewer scans (less than 7) are conducted. The results of energy dispersive X-ray spectrometry (EDS) (Carl Zeiss AG Corporate, Oberkochen, Germany) of the silver lines fabricated from these two reactive solutions are shown in Figure 5c. It can be seen that there is more silver in the deposit made with using the propylene glycol solution than in the one made with ethylene glycol solution. Therefore, the propylene glycol based processing solution yields silver lines with better physical properties. However, the propylene glycol based solution changes color to light yellow and then to gray within one hour of mixing at room temperature, while the ethylene glycol based solution is very stable for days. The change of color for the propylene glycol mixed solution indicates the presence of silver particles, and this was confirmed with UV-Vis spectrometry (*cf.* Figure 1b) which showed a strong absorption peak between 400 and 550 nm that corresponds to the presence of silver nano/micro particles [25]. These changes in optical and chemical properties have a serious effect of the stability of silver pattern synthesis, and so we used ethylene glycol based solutions for the remainder of our investigation in this study.

It should be noted that the LDSP process is in the liquid surroundings, and thermally induced flow with chemical reactions greatly affects the growth rate, morphology and uniformity of the silver line surface, especially in the early laser scans where the processing temperature is high. Therefore, the variation in the electrical resistance is large for silver line with small number of laser scans. With increasing the number of laser scans, the part that with thinner silver layer thickness would absorb more laser light owing to its smaller reflectivity than the thicker part. This phenomenon could therefore be used to self-adjust the thickness of the resulted silver lines with more laser scans. Nevertheless, the rough nanostructured surface of the silver lines could be useful for applications where a thin layer of materials with a large surface area is preferred. For example, in the electrodes for dye-sensitized and organic solar cells, a nanostructured electrode could significantly improve the conversion efficiency [26]. Furthermore, from Figure 5c, it is seen that the oxidation of the silver line fabricated by LDSP is not significant. This result could be attributed to the fact that LDSP is processed in a reducing environment where the reactive ion solution contains EG and formic acid. From the measured results of line thickness and resistance shown in Figure 5, the silver line fabricated with EG to ink solution of 1:1, 200 mW laser power and 10 scans is approximately 7.5 μΩ·cm.

**Figure 6:**
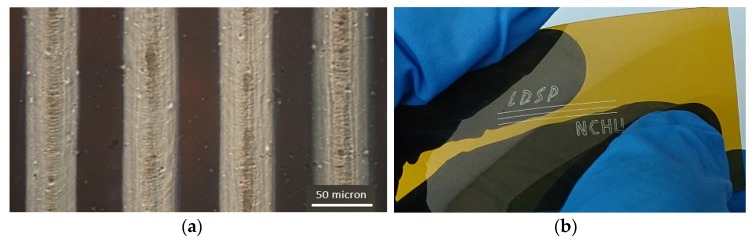
Silver lines fabricated on polyimide film substrate using LDSP (with ethylene glycol (EG) based solution): (**a**) microscopic picture of the silver line; (**b**) a demonstration of silver patterns on the flexible PI substrate.

Figure 6 shows the resulted silver lines and a demonstration of silver patterns on flexible polyimide (PI) film made by LDSP with the EG based processing solution. Note that the width of silver line in this study was about 50 μm, which will be used in the later analysis. It is emphasized again that this LDSP technique does not require photomasks or nanoparticles. Silver patterns in any shape can be rapidly fabricated on polymer substrates. During the LDSP process, silver patterns with micro porous may form as was shown in Figure 4. The liquid processing solution could fill and mend those pores/voids under subsequent laser scanning. Adding solvent with high boiling point such as ethylene glycol helps to minimize vaporization of the aqueous ammonium hydroxide based ion solution. Therefore, using appropriate processing solution and well controlling processing parameters are critically important to attain good electrical and mechanical properties in the LDSP process. To further understand the transport phenomena during the LDSP process, we conducted numerical analysis and will discuss the results in the following section.

### 4.2. Numerical Simulation Results

**Figure 7:**
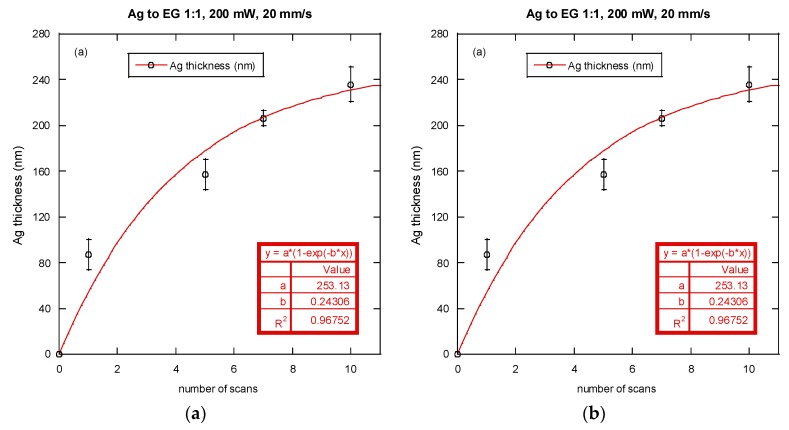
(**a**) Thickness of the silver film; (**b**) Reflectivity for 532 nm wavelength light *versus* number of laser scans (symbols: experimental data, lines: fitted curves).

The variations on the silver line thickness and reflectivity of the silver film with respect to the number of laser scans are shown in Figure 7. For each number of laser scan, five samples were prepared for measurement. Noted again that a white light interferometer was used to measure the film thickness and a hemispherical radiative property measurement system was used to measure the reflectivity as previously stated. A silver ion reactive solution using ethylene glycol at a ratio of 1:1 (volumetric) was used, the laser power was fixed at 200 mW and scanning speed was 20 mm/s. The curves shown in Figure 7a,b represent the fitted results for silver film thickness and reflectivity. These fitted curves have a coefficient of determination (*R*^2^) higher than 0.96 which indicates a reasonably good fit to the data. The initial reflectivity of the polyimide substrate at 532 nm was about 0.075. This goes up with the number of laser scans as the thickness of the silver film increases, and saturates at about 0.68 when the number of scans reaches 10. From these fitted results, a correlation between the reflectivity and the silver film thickness was obtained as:

(2) R=0.075+0.74(1−exp(−0.0072*δ))

**Figure 8:**
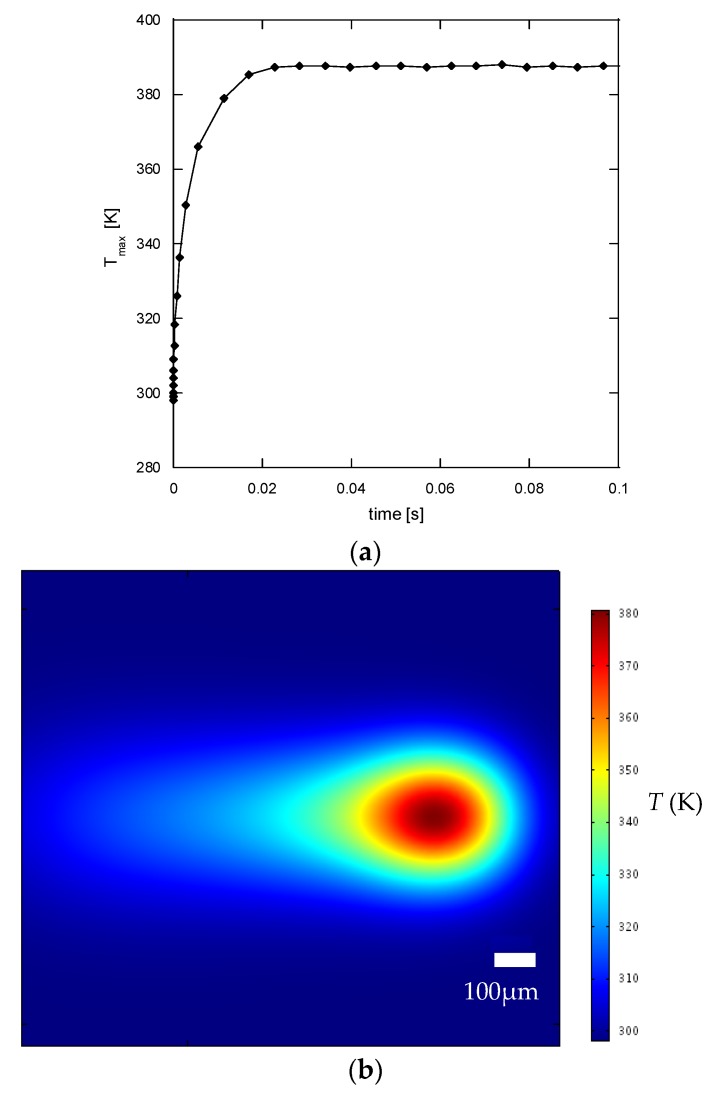
(**a**) The processing temperature for the first scan with respect to time along the scan path; (**b**) The simulated temperature profile on the polyimide surface.

Using the curve-fitted silver film reflectivity in Equation (2), the peak temperature at the center of the moving laser spot, defined as the processing temperature *T_p_*, was calculated from the numerical simulation for each laser scan. Figure 8a shows the processing temperature for the first scan with respect to time in the scan path (with a scan speed of 20 mm/s). It can be seen that the processing temperature reaches a steady value of approximately 387 K at a location 0.4 mm from the starting point. Figure 8b shows the simulated temperature profile on the polyimide surface. These results indicate a typical quasi-static thermal field for a moving heat source at constant speed and intensity [24]. Since the length of the scanned silver line was 10 mm, and the thickness of the silver was measured in a region near the center (~5 mm from the start), the quasi-static maximum temperature was adopted as the representative processing temperature.

The change in silver film thickness between each pair of consecutive scans (Δ*ξ*) was determined from Figure 7a, and the growth rate of the silver film ($\dot{r}$) was determined using the following equation:

(3) $$\dot{r}=\frac{\Delta \;\xi \;}{\tau_{ch}}$$

where the characteristic time ($\tau_{ch}$) is defined as:

(4) $$\tau_{ch}=\frac{silver\;line\;width\;}{speed\;of\;laser\;scanning}$$

**Figure 9:**
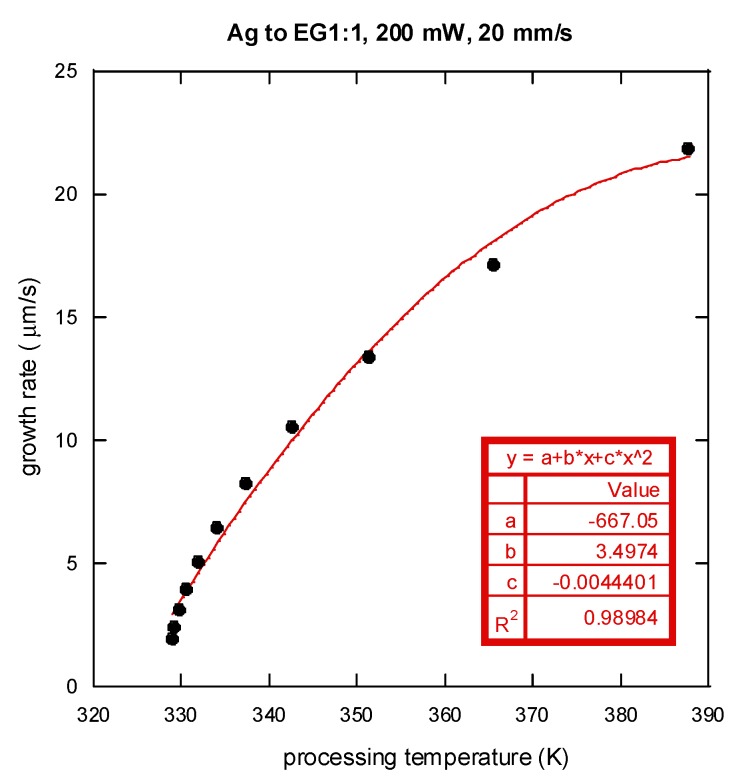
Variation of the silver line growth rate (in thickness) with respect to the processing temperature. The filled dots: the filled dots shown here were obtained using the fitted curve in Figure 7a.

Figure 9 shows the variation of film growth rate with respect to the processing temperature. A correlation of film growth rate and the processing temperature (*T_p_*) was then proposed:

(5) $$\dot{r}=-667+3.5T_{p}-0.0045{T_{p}}^{2}(T_{p}\text{ unit: K})$$

**Figure 10:**
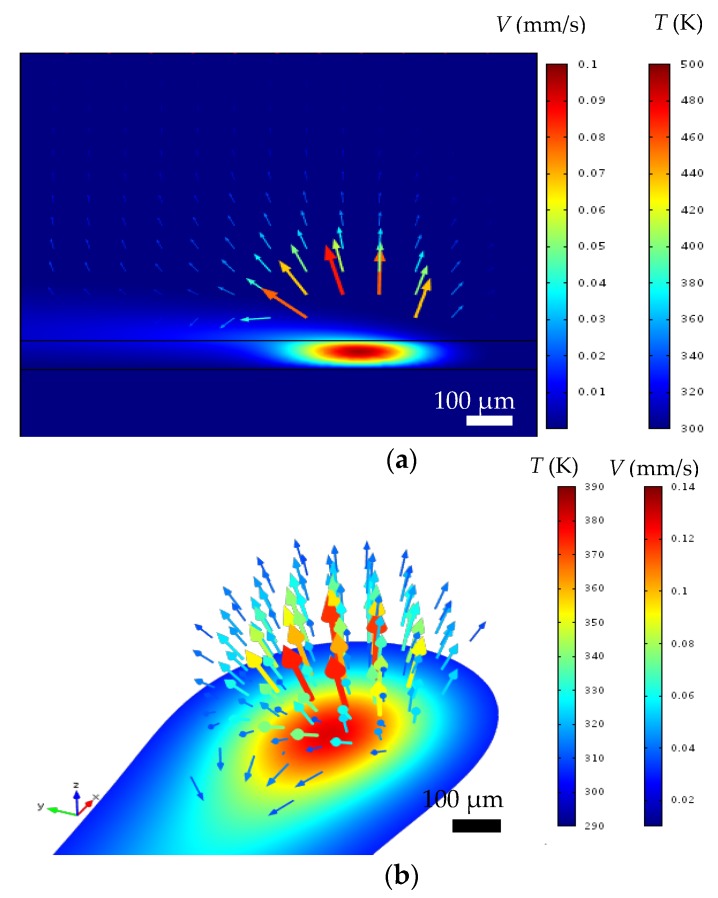
The velocity (arrows) and temperature (color surface) distribution near the laser focal spot, (**a**) Side view; (**b**) 3D view.

It should be noted that the filled dots in Figure 9 were obtained using the fitted curve in Figure 7a. From Figure 9, it can be seen that the growth rate of the silver film increases with a rise in temperature, which increases the rate of silver ion reduction. However, the increase in the silver growth rate with temperature then slows down at high temperature. It should also be noted that the growth rate cannot be adequately described by an Arrhenius type of reaction rate model where the growth rate would then increase exponentially with a rise in temperature. This is possibly due to the fact that the current LDSP process is in a liquid fluidic environment and during the pattern writing process, there is strong convective heat and mass transport near the focused laser spot on the substrate. Figure 10 shows the velocity and temperature distribution near the laser focal spot. There is significant fluid flow due to a strong buoyancy effect, while the heat for chemical reaction is confined in the vicinity above the substrate surface. As a result, the rate of silver deposition on the substrate may be adversely affected by the thermally-induced convective flow away from the substrate surface, the magnitude of which increases with a rise in temperature.

To illustrate the usefulness of the current analysis and proposed model for estimating the growth rate of the silver film fabricated by LDSP, simulations for a laser scanning speed of 30 mm/s were made to determine the film thickness after each scan. The computed processing temperature, silver film growth rate, film reflectivity and the accumulated silver film thickness are listed in Table 1. The computed silver line thickness for five, seven and 10 laser scans were compared with experimental results. It can be seen that the computed silver film thickness in each case is close to the measured results. However, it should be noted that the current analysis is valid for an LDSP process carried out within the temperature range investigated, *i.e.*, between 350 and 380 K. Working outside this range might result in the temperature being too low for the formation of a silver film or so high that the substrate would suffer thermal damage. It should also be emphasized that these results and the methodology presented here comes from the first investigation of transport characteristics of a LDSP process. The growth rate of the silver film indeed depends upon coupled thermo-fluidic transport with a chemical reaction, and can be regulated by adjustment of these parameters. It is shown that the proposed semi-empirical model can predict the silver film growth rate in an LDSP process using the parameters of laser power, scanning speed and number of scans. Furthermore, owing to the increased reflectivity of the silver film as its thickness increases, the growth rate decreases gradually to about 10 nm per laser scan after ten scans. It should be noted that this self-controlling effect corresponding to the coupled optical properties and reaction rate of LDSP process can be utilized to control the thickness and uniformity of the metal film.

It should be noted that, based on the experimental and numerical results for the LDSP process, to attain a growth rate higher than 10 μm/s, the processing temperature should be higher than 340 K. In addition, the reactive solution contains a small amount of formic acid. Therefore, a suitable polymer substrate for applying the LDSP should be selected with taking these limitations into consideration. Furthermore, in the current configuration of the LDSP process, the laser wavelength should be selected so the substrate can absorb the laser energy effectively to provide the heat for chemical reaction. It should also be pointed out that the growth rate of the silver pattern in the current status is less than 25 μm/s and the maximum line thickness achieved is about 250 nm owing to the increased reflectivity of the formed silver lines to the irradiated laser light. This self-limiting effect could restrict the application of LDSP from fabricating electronic devices that require thick metal patterns. Methods to improve the LDSP technique to address these aforementioned limitations are currently under investigation.

**Table 1:** Evaluation for the proposed model for laser direct synthesis and patterning (LDSP) process analysis processing parameters: (laser power: 200 mW, scanning speed: 30 mm/s, processing solution: ethylene glycol based solution (at a volumetric ratio 1:1).

Number of Scans	Reflectivity (Fitted Curve in Figure 7b)	Processing Temperature (k) (Numerical Results)	Growth Rate (μm/s) (Equation 3)	Accumulated Silver Film Thickness (nm) [ ]: Experimental Results
1	0.075	373.5	19.9	33.1
2	0.23	360.4	16.7	61.0
3	0.34	351.6	13.8	83.9
4	0.41	345.7	11.4	102.9
5	0.46	341.4	9.5	118.7 [150.7 ± 12.9]
6	0.50	338.5	8.1	132.2
7	0.53	336.1	6.9	143.6 [152.7 ± 10.9]
8	0.55	334.4	6.0	153.6
9	0.57	333.0	5.3	162.3
10	0.58	331.8	4.6	170.0 [182.9 ± 15.5]

## 5. Conclusions

In this study, the effects of the type and concentration of the additive solvent on the properties and growth rate of silver film fabricated by LDSP technology were investigated experimentally. It was shown that the reactive solution mixed with propylene glycol, which has higher viscosity, yields silver films with better physical properties than that from the solution mixed with ethylene glycol. However, it was observed that significant amount of silver nano/micro particles form within an hour in the propylene glycol based solution. To ensure a stable result, the use of an ethylene glycol based processing solution is recommended. A numerical analysis was also carried out to investigate the coupled opto-thermo-fluidic transport phenomena and the effects on the silver film growth rate. A semi-empirical model for estimating the silver film growth rate of the LDSP process was developed based on the experimental and numerical results. The proposed model was used to predict the silver film growth rate at different laser scanning speeds. The numerical results agree reasonably well with the experimental results. The proposed empirical model and analysis are clearly useful for the evaluation of the process parameters for metal film growth rate in processes of this kind.
