# Erratum: Tsai, S.-L.; *et al*. The Coupled Photothermal Reaction and Transport in a Laser Additive Metal Nanolayer Simultaneous Synthesis and Pattering for Flexible Electronics. *Nanomaterials* 2016, *6*, 12

**DOI:** 10.3390/nano6040062

**Published:** 2016-04-07

**Authors:** Nanoterials Editorial Office

**Affiliations:** MDPI AG, Klybeckstrasse 64, CH-4057 Basel, Switzerland; nanomaterial@mdpi.com; Tel.: +41-61-683-77-34

Due to an error during production, the Figure 7b in the published paper [1] was incorrect. The correct figure is as follows:

We apologize for any inconvenience caused to readers or authors by these changes. The article will be updated and the original will remain available on the article webpage.

## Figures and Tables

**Figure 7:**
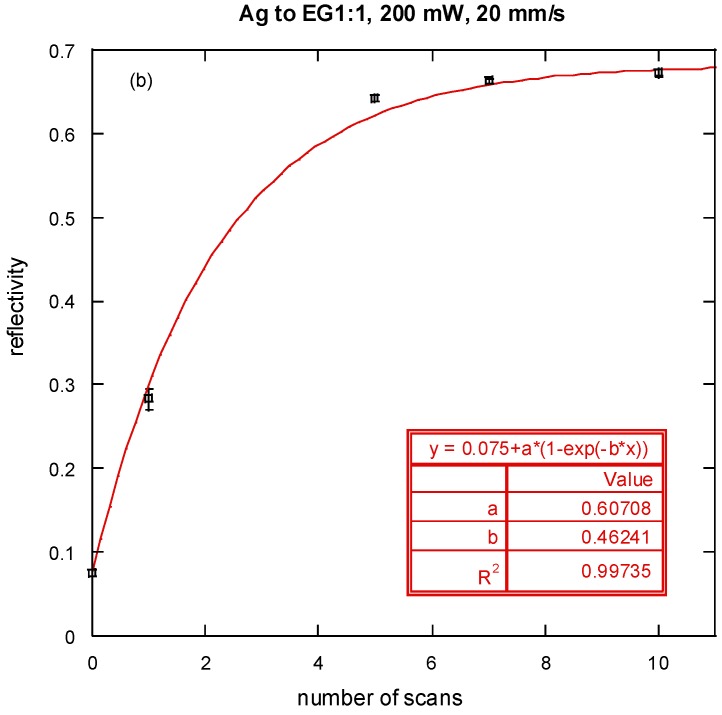
(**a**) Thickness of the silver film (**b**) reflectivity for 532 nm wavelength light *versus* number of laser scans (symbols: experimental data, lines: fitted curves).
